# Shear Behavior of Recycled Fine Aggregate Reinforced by Nano-MgO Modified Cement

**DOI:** 10.3390/ma15207188

**Published:** 2022-10-15

**Authors:** Ting Zhu, Yitong Shou, Xiaoqing Chen, Beifeng Lv, Xianwen Huang, Yanfei Yu, Cuihong Li

**Affiliations:** 1 School of Civil Engineering, Shaoxing University, Shaoxing 312000, China; 2 Shaoxing City Investment Renewable Resources Co., Ltd., Shaoxing 312000, China; 3 Shaoxing City Investment Group Ltd., Shaoxing 312000, China; 4 School of Civil Engineering, Suzhou University of Science and Technology, Suzhou 215009, China; 5 Shaoxing Key Laboratory of Interaction between Soft Soil Foundation and Building Structure, Shaoxing 312000, China

**Keywords:** recycled fine aggregate, nano-MgO, direct shear test, triaxial shear test

## Abstract

In order to study the mechanical modification effect of nano-MgO on cement-reinforced recycled fine aggregate (CRA), direct shear tests and triaxial shear tests were carried out. In the test of recycled fine aggregate reinforced by nano-MgO modified cement (MCRA), the cement content was fixed at 2%, and the nano-MgO content varied between 0%, 0.5%, 1.0%, 1.5% and 2.0%. The test results showed that adding nano-MgO can greatly increase both the direct shear strength and triaxial shear strength of MCRA. This increase in direct shear strength was mainly attributed to the increase in cohesion. However, this increase in triaxial shear strength was attributed to the simultaneous increase in the cohesion and friction angle.

## 1. Introduction

The progress of urban development in China has led to the demolition of old buildings, resulting in a large amount of construction demolition waste [1]. In the last decade, 2.36 billion tons of construction waste has been generated yearly [2]. Generating such a large amount of construction waste without proper management will have adverse environmental impacts, including landfill depletion, greenhouse gas emissions, and water pollution [3,4]. In order to implement the concept of sustainable development, the Ministry of Transport encourages the recycling utilization of waste roads, construction waste, and industrial solid waste [5].

Recycled aggregate is a building material in construction waste that can be recycled through various processing techniques. Its recycling can not only alleviate the shortage of natural aggregate, but also help to manage construction waste [6,7]. In the treatment and application of recycled aggregates, different curing materials are usually added to improve their mechanical properties [8,9,10], among which nanomaterials have been widely used due to their excellent properties [11,12,13]. Bibhuti et al. [14] measured the compressive strength of all-natural and recycled aggregate concrete mixtures with or without nano-SiO_2_ at 7, 28, 90, and 365 days. The research results showed that the replacement of natural aggregate with recycled aggregate had a significant effect on the compressive strength of concrete; the compressive strength and microstructure of concrete were improved with the incorporation of nano-SiO_2_. In order to further study the effect of nano-SiO_2_ on the properties of recycled aggregates, Nuaklong et al. [15] explored the improvement of the mechanical properties and durability of recycled aggregate concrete using nano-SiO_2_. The experimental results showed that adding 1% nano-SiO_2_ could increase the compressive strength of recycled aggregate by 13%, and the porosity of the structure could be effectively reduced. Zeng et al. [16] immersed recycled aggregate in nano-SiO_2_ suspension for modification and found through a compressive strength test and microhardness test that nano-SiO_2_ could effectively increase the compressive strength of recycled aggregate; additionally, durability improved and had stronger resistance to corrosion and less corrosion-induced cracks. Qu [17] studied the strengthening effect of nano-CaCO_3_ on the mechanical properties of recycled aggregate concrete. By performing a compressive strength test and a flexural strength test, it was found that nano-CaCO_3_ could significantly improve the compressive strength and flexural strength of recycled aggregate. To a certain extent, the mechanical properties can exceed those of ordinary concrete. Nanomaterials not only have a significant effect on the mechanical properties of recycled aggregates, but also have a good effect on improving its pore structure. Ying et al. [18] incorporated nano-SiO_2_ and nano-TiO_2_ into recycled aggregate concrete. By implementing the molecular imprinting method, it was found that nano-SiO_2_ and nano-TiO_2_ could achieve the filling effect by refining the cross area in cement and producing denser concrete, which made the overall structure of recycled aggregate concrete denser. The improvement effect of both materials was the best with a content of 2%. Within the literature, the application of nanomaterials such as nano-SiO_2_, nano-CaCO_3_ as well as nano-TiO_2_ in recycled aggregates is extensive; however, the application of nano-MgO to recycled fine aggregates is relatively rare.

Therefore, in this paper, nano-MgO was used to modify the shear behavior of cement-reinforced recycled fine aggregate (CRA), and the effect of nano-MgO content on the mechanical properties of MCRA was explored by performing direct shear and triaxial shear tests. The recycled fine aggregate reinforced by nano-MgO modified cement can be used as road bases or base fillers, so as to achieve the engineering reuse of recycled aggregate; realize its engineering value repeatedly; maximize environmental protection and resource conservation; and make important contributions to sustainable development.

## 2. Test Materials and Methods

### 2.1. Materials

The recycled aggregate used in this test was collected from a construction site in Erhuan North Road, Shaoxing City, Zhejiang Province, China. After removing the asphalt layer on the abandoned road subgrade, the aggregate at the bottom of the subgrade was crushed and screened to obtain recycled fine aggregate with a particle size of less than 4.75 mm. Laboratory geotechnical tests of recycled fine aggregates were carried out according to “Test Methods of Materials Stabilized with Inorganic Binders for Highway Engineering” (JTG E51-2009) [19], and their basic physical properties were measured as shown in Table 1, which was obtained from the research of Lv et al. [20]. The main chemical components of recycled fine aggregate are SiO_2_ and CaCO_3_. The cement used in the test was PO32.5 ordinary Portland cement, and the basic physical properties are shown in Table 2, which was sourced from the research of Lv et al. [21]. The nano-MgO used in the test was produced by Shanghai Macklin Biochemical Co., Ltd. (Shanghai, China), model M813080, and was obtained in the form of white powder. The specific physical parameters are shown in Table 3, which is from the research of Wang et al. [22]. In order to meet the actual application of the project, the test water is ordinary laboratory tap water composed of water and some ions, such as calcium, magnesium, and potassium.

### 2.2. Test Plan

In this test, the recycled aggregate of waste road was used as the basic soil sample; then, cement and nano-MgO were mixed to carry out a direct shear and a triaxial test, respectively. Each test was divided into 5 groups with 4 samples in each group. The specific test plan is shown in Table 4. The cement content is the mass ratio of cement to dry recycled aggregate, the nano-MgO content is the mass ratio of nano-MgO to dry recycled aggregate, and the moisture content is the mass ratio of water to the mixture. In Table 4, MCRAY represents the nano-MgO cement-reinforced recycled aggregate, and Y represents the content of nano-MgO.

### 2.3. Sample Preparation

#### 2.3.1. Direct Shear Sample

The preparation process of the direct shear test samples can be mainly divided into the following steps:

(1) The recycled aggregate of waste road was placed into an oven to dry; then, the large particles and sundries were screened by using a standard sieve with a pore size of 4.75 mm.
(2) According to the mixing ratio, the test materials including cement, recycled aggregate, nano-MgO and water were weighed and stirred with a mixer for 10 min to ensure the mixture was fully and uniformly stirred.
(3) Vaseline was evenly smeared on the inner wall of the ring mold with a diameter of 61.8 mm and a height of 20 mm. The fully stirred mixture was then poured into the mold and compacted.
(4) The prepared samples were cured for 7 days; then, the mold was removed for testing.

#### 2.3.2. Triaxial Shear Sample

The preparation process of the triaxial shear test samples can be divided into the following steps:

(1) The recycled aggregate of waste road was placed into an oven to dry; then, the large particles and sundries were screened by using a standard sieve with a pore size of 4.75 mm.
(2) According to the mixing ratio, the test materials mentioned above were weighed and stirred with a mixer for 10 min to ensure the mixture was fully and uniformly stirred. The fully stirred mixture was then poured into the mold three times and in compacted in layers.
(3) A cylindrical sample with a diameter of 39.1 mm and a height of 80 mm was made by using a mold; it was then cured for 7 days.

**Figure 1:**
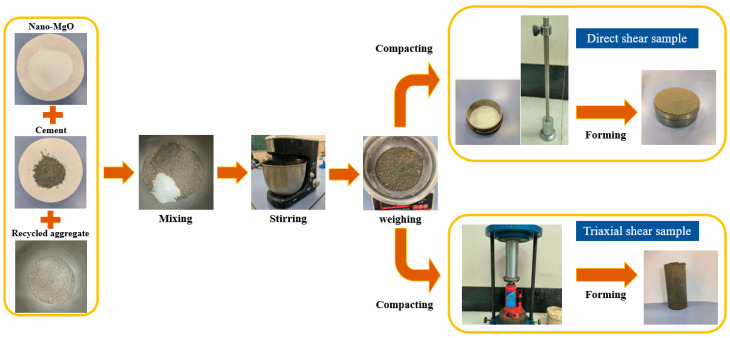
Sample preparation process.

The sample preparation process for direct shear and triaxial shear tests is shown in Figure 1.

### 2.4. Test Methods

#### 2.4.1. Direct Shear Test

The SJ-1 strain-controlled direct shear instrument produced by Zhejiang Tugong Instrument Co., Ltd. (Shaoxing, China) was adopted in the direct shear test. This test is designed as an unconsolidated quick shear test. During the test, the prepared sample was placed into the direct shear box, and the normal stress applied to the sample was controlled at 100, 200, 300, and 400 kPa, respectively. The shear rate was set to 0.8 mm/min, and the reading of the proving ring was recorded every 15 s. After the test, the shear stress and shear displacement were calculated from the recorded data.

#### 2.4.2. Triaxial Shear Test

The fully automatic triaxial tester TKA-TTS-3S produced by Nanjing TKA Technology Co., Ltd. (Nanjing, China) was used in the triaxial shear test. This test is designed as an unconsolidated and an undrained shear test. During the test, the samples were placed on a metal test table with a smooth bottom, and an epoxy resin indenter was placed on the upper end. The confining pressures were controlled at 100, 200, 300, and 400 kPa, respectively. The shear rate was set to 1 mm/min, and the test was stopped when the axial strain reached 20%.

## 3. Direct Shear Test Results and Analysis

### 3.1. Shear Displacement-Stress Curve

**Figure 2:**
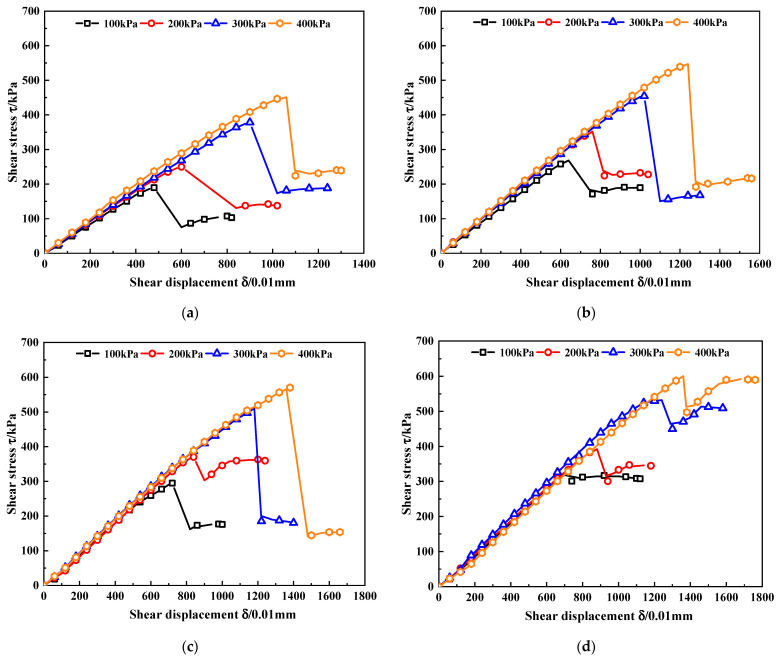

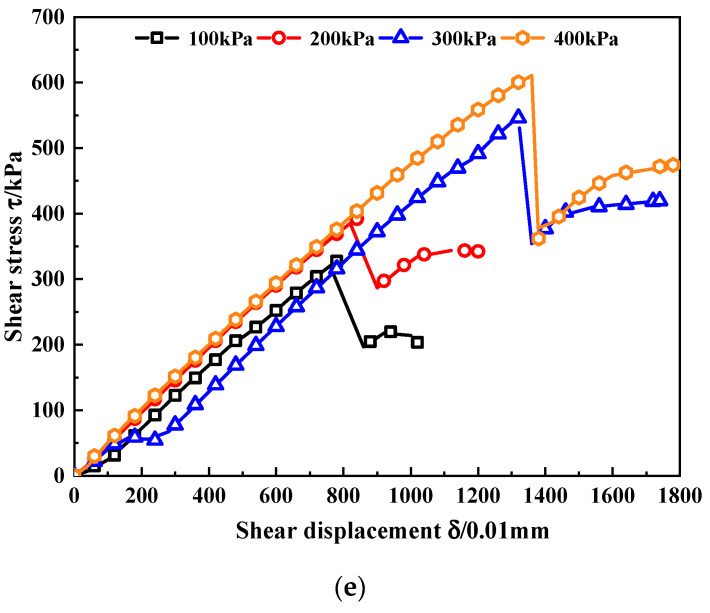
Shear displacement-stress curves of MCRA. (**a**) MCRA0. (**b**) MCRA0.5. (**c**) MCRA1.0. (**d**) MCRA1.5. (**e**) MCRA2.0.

The shear displacement-stress curves of MCRA with different nano-MgO contents obtained through the direct shear tests are shown in Figure 2. With the increase in shear displacement, the shear stress also increases stably. After reaching the peak stress, the shear stress decreases rapidly, and the rebound phenomenon occurs. At the same time, the larger the normal stress and the higher the nano-MgO content are, the larger the shear displacement required for MCRA to reach the peak stress.

### 3.2. Peak Stress

**Figure 3:**
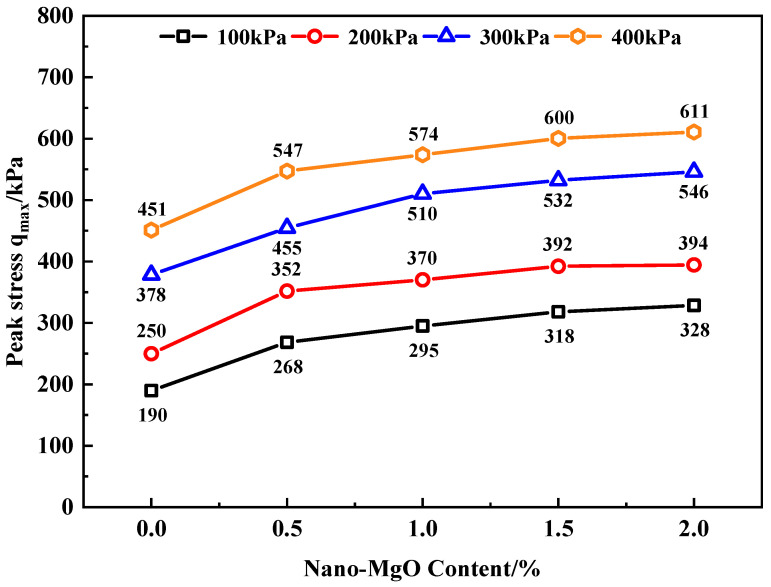
The relationship between peak stress and normal stress of MCRA.

According to the shear displacement-stress curve of MCRA shown in Figure 2, the peak stress of MCRA with different nano-MgO contents is obtained, and the relationship between the peak stress of MCRA and the normal stress is drawn, as shown in Figure 3.

It can be found from Figure 3 that the peak stress (shear strength) of MCRA increases stably with an increase in the normal stress under the same nano-MgO content. When the normal stress increases from 100 to 200 kPa, the increase in the peak stress of each group of MCRA remains stable between 20% and 31%. When the normal stress increases from 200 to 300 kPa, the peak stress of MCRA increases from 29% to 51%, and the growth rate is more obvious. When the normal stress increases from 300 to 400 kPa, the peak stress of MCRA increases between 12% and 20%, and the growth rate is small. Therefore, when the normal stress increases from 200 to 300 kPa, the increase in the peak stress is the largest; when the normal stress increases from 300 to 400 kPa, the increase in the peak stress is the smallest.

Under the same normal stress, with an increase in nano-MgO content, the shear strength of MCRA also increases, and the growth trend is basically the same under each normal stress. When the nano-MgO content is 0.5%, the shear strength of MCRA0.5 increases significantly. When the normal stress is 100, 200, 300 and 400 kPa, the shear strength of MCRA0.5 increases by 41%, 41%, 20% and 21%, respectively, compared with that of MCRA0, indicating that the incorporation of nano-MgO can greatly increase its shear strength. This is mainly because that nano-MgO will undergo Pozzolanic reaction, whereby the hydration product Mg(OH)_2_ of MgO and C-S-H gel of cement are tightly bounded together. In the meantime, volume expansion will occur during the conversion of MgO to Mg(OH)_2_, which can fill the pores and improve the shear strength of the sample [23,24]. In the subsequent process of increasing the nano-MgO content, the increase in the shear strength begins to slow down. When the nano-MgO content increases from 0.5% to 1.0% and from 1.0% to 1.5%, the increase in the shear strength of MCRA is stable between 4% and 12%. Finally, when the nano-MgO content increases from 1.5% to 2.0%, the increase in the shear strength is only between 1% and 3%, with a slight increase.

When the normal stress is 100 kPa, the shear strength of MCRA2.0 reaches 328 kPa, which is 73% higher than that of MCRA0 of 190 kPa. When the normal stress is 200 kPa, the shear strength of MCRA2.0 is 394 kPa, which is 58% higher than that of MCRA0. When the normal stress is 300 kPa, the shear strength of MCRA2.0 is 546 kPa, which is 44% higher than that of MCRA0. When the normal stress is 400 kPa, the shear strength of MCRA2.0 sample is 611 kPa, which is 35% higher than that of MCRA0. Therefore, under the normal stress of 100 kPa, with the increase in nano-MgO content, the shear strength of MCRA increases the most obviously, while the shear strength increases the least under the normal stress of 400 kPa.

### 3.3. Shear Strength Indexes

In order to better compare the shear strength of the samples, the shear strength of MCRA was fitted linearly with the normal stress to obtain its strength curve. The shear strength index of MCRA can be obtained from the strength curve, as shown in Table 5.

Table 5 shows that with the increase in nano-MgO content, the internal friction angle *φ* and cohesion *c* of MCRA gradually increase, but the increase in the internal friction angle is relatively small. Compared with MCRA0, the internal friction angle of MCRA 0.5, MCRA1.0, MCRA1.5 and MCRA2.0 only increases by 2%, 5%, 5%, and 6%, respectively; thus, the increase in the internal friction angle is not obvious. Compared with the change of the internal friction angle, the cohesion of the samples changes sharply with the increase in nano-MgO content. Compared with MCRA0, the cohesion of MCRA0.5, MCRA1.0, MCRA1.5 and MCRA2.0 increases by 92%, 117%, 140%, and 147%, respectively, with an obvious increase. It can also be found that the incorporation of 0.5% nano-MgO into CRA greatly improves its cohesion. When the nano-MgO content increases from 0.5% to 1.0% and 1.0% to 1.5%, the cohesion increases by 13% and 11%, respectively, and the growth rate slows down. Finally, the cohesion of MCRA2.0 increases by 3% compared with that of MCRA1.5, with slight improvement. The overall change trend of the cohesion is highly consistent with the change law of the shear strength of MCRA. Therefore, it can be considered that during the direct shear test, nano-MgO mainly improved the shear strength of MCRA by increasing its cohesion.

## 4. Triaxial Shear Test Results and Analysis

### 4.1. Deviatoric Stress–Strain Curve

**Figure 4:**
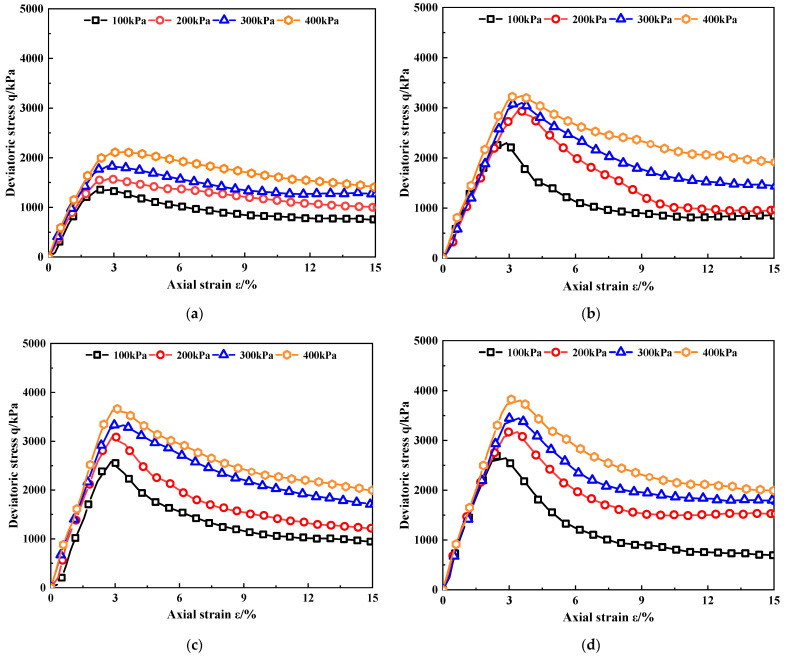

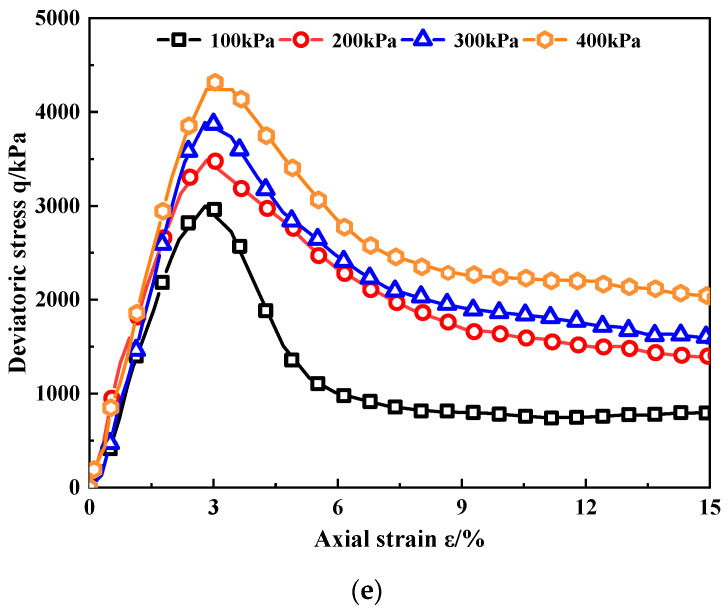
Deviatoric stress–strain curves of MCRA. (**a**) MCRA0. (**b**) MCRA0.5. (**c**) MCRA1.0. (**d**) MCRA1.5. (**e**) MCRA2.0.

Figure 4 shows the relationship between the deviatoric stress q of MCRA and the axial strain ε (hereafter referred to as the deviatoric stress–strain curve). It can be seen from Figure 4 that the deviatoric stress–strain curves of MCRA are all softening curves. With an increase in the strain, the stress shows an obvious downward trend, and with an increase in confining pressure, the softening trend gradually weakens. However, with the increase in nano-MgO content, the softening characteristics became more obvious. According to the “Standard for Geotechnical Testing Method” (GB/T 50123-2019) [25], the maximum axial strain should be from 3% to 5% greater than the failure strain or the axial strain when the deviatoric stress tends to be stable. In this study, 15% is taken as the maximum axial strain.

### 4.2. Peak Stress

**Figure 5:**
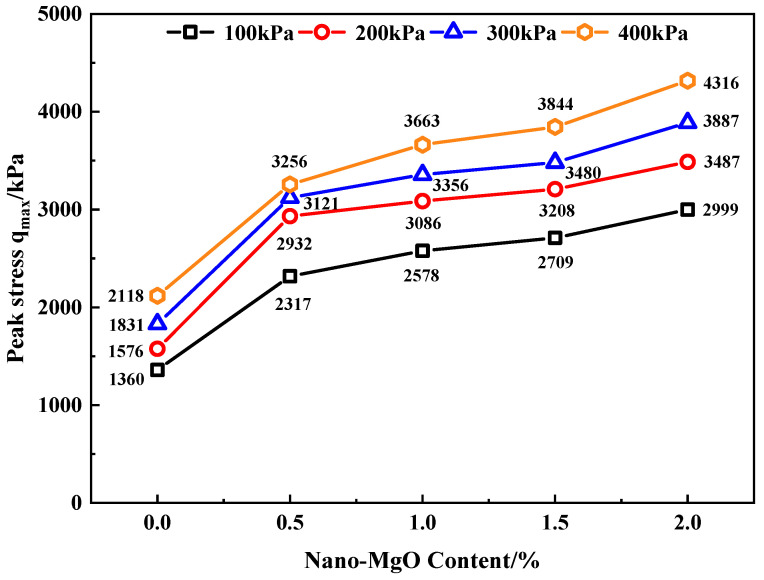
The relationship between peak stress and confining pressure of MCRA.

By analyzing Figure 4, the peak stress *q*_max_ of MCRA corresponding to different nano-MgO contents can be obtained, and the relationship between the peak stress and confining pressure of MCRA can be found, as shown in Figure 5.

It can be found from Figure 5 that the peak stress of MCRA increases with the increase in confining pressure when the nano-MgO content is fixed. When the confining pressure increases from 100 to 200 kPa, the increase in the peak stress of MCRA in each group is stable between 16% and 27%, with an obvious increase. When the confining pressure increases from 200 to 300 kPa, the peak stress of MCRA increases in the range of 6% to 16%. When the confining pressure increases from 300 to 400 kPa, the peak stress of MCRA increases between 4% and 16%. Therefore, under low confining pressure (that is, when the normal stress increases from 100 to 200 kPa), the increase in peak stress is the highest. When the normal stress increases from 200 to 300 kPa and 300 to 400 kPa, the increase in peak stress is smaller and there is little difference between the two groups of confining pressures.

Under the same confining pressure, the shear strength of MCRA increases significantly with the increase in nano-MgO content, and the shear strength shows the same increasing trend under each confining pressure. Firstly, when the nano-MgO content is 0.5%, the shear strength of MCRA increases sharply. When the confining pressure is 100, 200, 300, and 400 kPa, the shear strength of MCRA0.5 increases by 70%, 86%, 70%, and 54%, respectively, compared with that of MCRA0. It also shows that the incorporation of nano-MgO into CRA can greatly increase its shear strength, which is the same as that obtained in the direct shear test. The growth of the shear strength of MCRA shows a slowing trend when the nano-MgO content increases continuously. When the nano-MgO content increases from 0.5% to 1.0% and from 1.5% to 2.0%, the increase in the shear strength of MCRA is stable between 5% and 13%. Meanwhile, when the nano-MgO content increases from 1.0% to 1.5%, the increase in the shear strength is only from 4% to 5%, which is relatively small.

When the confining pressure is 100 kPa, the shear strength of MCRA2.0 is 2999 kPa; when the confining pressure is 200 kPa, the shear strength of MCRA2.0 is 3487 kPa, with that of MCRA0 being 1576 kPa; thus, they both increase by 121%. When the confining pressure is 300 kPa, the shear strength of MCRA2.0 reaches 3887 kPa, which is 112% higher than that of MCRA0; when the confining pressure is 400 kPa, the shear strength of MCRA2.0 is 4316 kPa, which is 104% higher than that of MCRA0. Under the four confining pressures, the shear strength of MCRA2.0 is significantly improved. It can be concluded that under the confining pressures of 100 kPa and 200 kPa, with the increase in nano-MgO content, the peak strength of MCRA has the largest increase. The peak strength of MCRA increases the least at 400 kPa, which is also basically consistent with the conclusion of the direct shear test.

### 4.3. Strength Curves and Shear Strength Parameters

**Figure 6:**
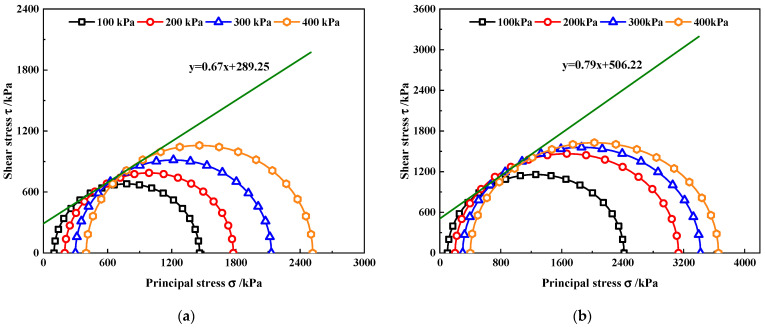

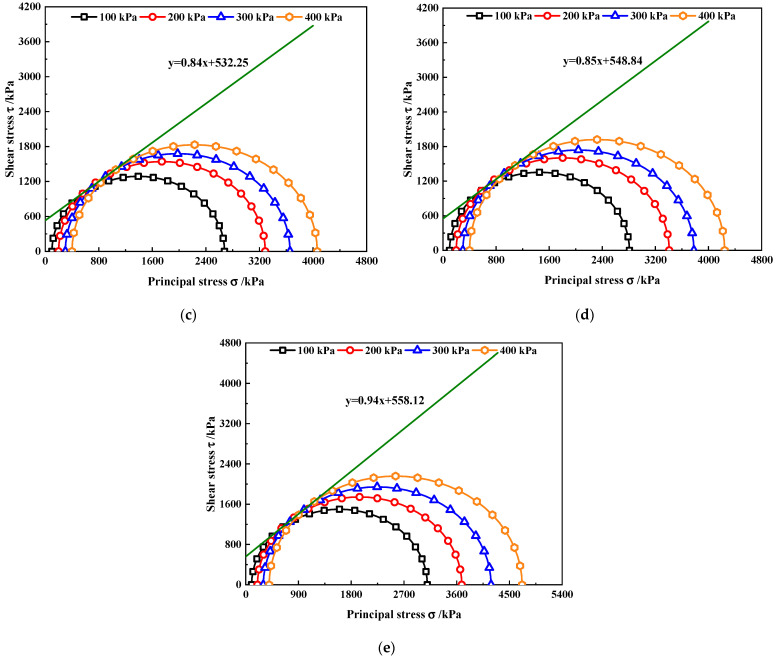
Moiré failure stress circle envelopes of MCRA. (**a**) MCRA0. (**b**) MCRA0.5. (**c**) MCRA1.0. (**d**) MCRA1.5. (**e**) MCRA2.0.

According to the deviatoric stress–strain curve, the Moiré failure stress circle under different nano-MgO contents was obtained, and the common tangent of the Moiré circle under four different confining pressures was used as the shear strength envelope of the sample, as shown in Figure 6. Meanwhile, the shear strength formula, internal friction angle (*φ*) and cohesion (*c*) were obtained from the shear strength envelope, as shown in Table 6.

It can be seen from Table 6 that with the increase in nano-MgO content, the internal friction angle and cohesion of MCRA gradually increases, and that of MCRA2.0 reaches the maximum values, which are 43.26° and 558.12 kPa. The internal friction angles of MCRA0.5, MCRA1.0, MCRA1.5 and MCRA2.0 increase by 13%, 17%, 19%, and 27%, respectively, compared with that of MCRA0. Compared with the change law of the internal friction angle in the direct shear test, it has a relatively obvious increase. The cohesion of MCRA0.5, MCRA1.0, MCRA1.5 and MCRA2.0 increases by 75%, 84%, 90%, and 93%, respectively, compared with that of MCRA0. However, it is slightly lower than the increase in cohesion in the direct shear test. When the nano-MgO content increases from 0.5% to 1.0%, the internal friction angle and cohesion increase by 4% and 5%, respectively; when the nano-MgO content increases from 1.0% to 1.5%, the internal friction angle and cohesion increase by 2% and 3%, respectively. Finally, the internal friction angle and cohesion of MCRA2.0 increase by 7% and 2%, respectively, compared with that of MCRA1.5. It can be found that the internal friction angle and cohesion have the same change law as the shear strength of MCRA. Therefore, it can be concluded that in the triaxial test, nano-MgO can improve the shear strength of MCRA by simultaneously increasing the internal friction force and cohesion.

## 5. Conclusions

In this paper, nano-MgO was used to modify the mechanical properties of recycled aggregate reinforced by cement. By performing direct shear tests and triaxial shear tests, the influence of the nano-MgO content on the shear strength of recycled aggregate reinforced by cement is explored. The developed method of using recycling materials in this research provides important technical guidance for the realization of resource recovery and the recycling of renewable materials, and has important environmental protection and practical significance. 

In the direct shear test, it was found that the direct shear strength of CRA can be significantly improved by adding nano-MgO. The direct shear strength of MCRA increases with the increase in nano-MgO content. When the normal stress is 100, 200, 300 or 400 kPa, the direct shear strength of MCRA2.0 is 73%, 58%, 44% or 35% higher than that of MCRA0, respectively. When the content of nano-MgO increases from 0.5% to 2.0%, the internal friction angle increases only by 2%, 5%, 5% and 6%, respectively, while the cohesion increases by 92%, 117%, 140%, and 147%, respectively. Nano-MgO can improve the direct shear strength of MCRA by increasing its cohesion.

In the triaxial shear test, it was found that the addition of nano-MgO can significantly improve the triaxial shear strength of CRA. The triaxial shear strength of MCRA increases with the increase in nano-MgO content, and the test results are highly consistent with the direct shear test results. When the normal stress is 100, 200, 300 and 400 kPa, the triaxial shear strength of MCRA2.0 is 121%, 121%, 121%, and 104% higher than that of MCRA0, respectively. When the content of nano-MgO increases from 0.5% to 2.0%, the internal friction angle increases by 13%, 17%, 19%, and 27%, respectively, and the cohesion increases by 75%, 84%, 90%, and 93%, respectively. Nano-MgO can improve the triaxial shear strength of MCRA by simultaneously increasing its internal friction angle and cohesion.

## Figures and Tables

**Table 1:** Basic physical properties indexes of recycled aggregate [20].

Index	Moisture Content (%)	Plastic Index	Specific Gravity	Silt Content (%)	Apparent Density (kg/m^3^)
Value	12.9	16.6	2.68	20.5	2.67

**Table 2:** Physical performance indexes of PO32.5 cement [21].

Fineness (%)	Initial Setting Time (min)	Final Setting Time (min)	3 Days Compressive Strength (MPa)	28 Days Compressive Strength (MPa)	3 Days Flexural Strength (MPa)	28 Days Flexural Strength (MPa)
3.4	210	295	26.9	48.1	4.9	9.0

**Table 3:** Nano-MgO physical parameters [22].

Product	Average Particle Size (nm)	Product Purity (%)	Theoretical Density (g/cm^3^)	Melting Point (°C)	Boiling Point (°C)	Crystal Form	Dispersity
Nano-MgO	15–20	99.9	3.580	2850	3600	Near spherical	Preparation by gas phase method

**Table 4:** Test plan.

Sample No.	Cement Content (%)	Nano-MgO Content (%)	Normal Stress/Confining Pressure (kPa)	Moisture Content (%)	Curing Age (d)
MCRA0	2	0	100, 200, 300, 400	10	7
MCRA0.5		0.5			
MCRA1.0		1.0			
MCRA1.5		1.5			
MCRA2.0		2.0			

**Table 5:** Shear strength indexes of MCRA.

Sample No.	Strength Curve	Internal Friction Angle *φ*/°	Cohesion *c*/kPa
MCRA0	τ = 0.913x + 31.65	42.38	89.06
MCRA0.5	τ = 0.939x + 61.21	43.20	170.61
MCRA1.0	τ = 0.976x + 67.97	44.31	193.16
MCRA1.5	τ = 0.987x + 75.22	44.61	213.98
MCRA2.0	τ = 0.998x + 76.87	44.95	220.35

**Table 6:** Shear strength parameters of MCRA.

Sample No.	Strength Envelops	Internal Friction Angle *φ* (°)	Cohesion *c* (kPa)
MCRA0	τ = 0.67x + 289.25	33.98	289.25
MCRA0.5	τ = 0.79x + 506.22	38.32	506.22
MCRA1.0	τ = 0.84x + 532.25	39.89	532.25
MCRA1.5	τ = 0.85x + 548.84	40.53	548.84
MCRA2.0	τ = 0.94x + 558.12	43.26	558.12

## Data Availability

Not applicable.
